# Characterization of a Dihydromyricetin/α-Lactoalbumin Covalent Complex and Its Application in Nano-emulsions

**DOI:** 10.3390/foods12142783

**Published:** 2023-07-21

**Authors:** Ninghai Lu, Limin Wu, Shiyu Zhen, Benguo Liu

**Affiliations:** 1 School of Resource and Environment, Henan Institute of Science and Technology, Xinxiang 453003, China; gsninghai@163.com (N.L.); xxwu5@163.com (L.W.); 2 School of Food Science, Henan Institute of Science and Technology, Xinxiang 453003, China; jysyzhen@126.com

**Keywords:** dihydromyricetin, α-lactoalbumin, covalent complex, antioxidant activity, nano-emulsion

## Abstract

A dihydromyricetin (DMY)/α-lactoalbumin (α-La) covalent complex was prepared and characterized, and its application in nano-emulsions was also evaluated in this study. The results suggested that the covalent complex could be obtained using the alkaline method. The UV and IR spectra confirmed the formation of the covalent complex, and the amount of DMY added was positively correlated with the total phenol content of the complex. The complex had an outstanding 2,2′-azinobis(3-ethylbenzothiazoline-6-sulfonic acid) (ABTS)-radical-scavenging ability, reducing power and α-glucosidase inhibitory activity, which were positively related to its total phenol content. The complex could be used as an emulsifier to stabilize the β-carotene-loaded nano-emulsion. The stability and β-carotene-protective capacity of the nano-emulsion stabilized by the complex were also positively related to the total phenol content of the complex, being higher than those of the nano-emulsion developed using α-La. Our results provide a reference for the construction of a new food delivery system and extend the applications of α-La and DMY in foods.

## 1. Introduction

Proteins and polyphenols are important components of food. Proteins can stabilize emulsions and foams, enhance solution viscosity and form gels [1,2]. Polyphenols possess many biological functions, such as anti-tumor, anti-oxidation, anti-inflammation, and anti-diabetes activities, and so on [3,4]. Complexes can be formed between proteins and polyphenols through covalent bonds [5]. The most common method used to prepare a protein/polyphenol covalent complex is to mix the components in alkaline solution in the presence of oxygen [6]. During this process, the polyphenols are first oxidized to quinones and then react with free amino groups of lysine residues, cysteine residues and tryptophan residues in the protein to form covalent complexes [7]. Previous studies have shown that the protein/polyphenol covalent complex has an excellent antioxidant and emulsifying capacity and can be used as a carrier to transfer bioactive substances to specific sites so as to improve the bioavailability of bioactive substances [8]. Liu et al. found that a hemp protein isolate–gallic acid conjugate significantly improved the solubility, antioxidant activity and emulsifying properties of a hemp protein isolate [9]. Zhao et al. also found that the antioxidant performance of fish oil could be improved using anchovy protein hydrolysates–polyphenol conjugates [10].

α-Lactalbumin (α-La) is widely distributed in the milk of humans and other mammals (cattle, goats, camels, horses, etc.). It is the second highest protein in whey protein, accounting for 20–25% of the total whey protein. Because α-La is rich in tryptophan, cysteine, lysine and other essential amino acids, it has been widely applied in baby food, health products, medicine and other fields [11,12]. Dihydromyricetin (DMY) is a common flavonoid, which is present at a level of up to 30% in *Ampelopsis grossedentata* leaves (Tengcha tea) [13,14]. It has achieved industrial production and has wide application prospects. In a previous study, we investigated the non-covalent interaction between DMY and α-La and found that their interaction could improve the antioxidant activity and emulsifying ability of α-La [15]. In the process and storage of food, protein and flavonoids can also form a covalent complex, which has a significant effect on the nature and function of the food. In this study, the covalent combination of α-La and DMY was systematically evaluated. Firstly, the covalent complex of DMY and α-La was prepared using the alkaline method and characterized. Then, its antioxidant and α-glucosidase inhibitory capacities were analyzed. Finally, the complex was used to stabilize the β-carotene-loaded nano-emulsion.

## 2. Materials and Methods

### 2.1. Chemicals

α-La was obtained from the Davisco company (Le Sueur, MN, USA). Medium-chain triglyceride (MCT) and DMY were obtained as products of Yuanye Bio-Technology Co., Ltd. (Shanghai, China). β-Carotene, p-nitrophenyl-α-D-glucopyranoside (pNP-G), 2′-azino-bis(3-ethylbenzothiazoline-6-sulfonic acid) (ABTS) and α-glucosidase were purchased from Sigma-Aldrich (St. Louis, MO, USA). The other chemicals were of analytical grade.

### 2.2. Preparation and Characterization of the Covalent Complex

The α-La/DMY covalent complex was prepared based on the report of Liu et al. [16]. Then, 50 mL of 0.5% α-La solution was mixed with 50 mL DMY solution at different concentrations (0.02%, 0.05%, 0.1%, *w*/*w*). The pH value of the reaction system was adjusted to 9.0 with 0.5 M NaOH solution. After 24 h of reaction, it was dialyzed for 48 h. After freeze-drying, the covalent complexes of C-1, C-2 and C-3 were obtained. Their UV spectra in water (220–400 nm) were measured with a UV spectrophotometer, with DMY and α-La as controls. The FT-IR spectra (4000–400 cm^−1^) were measured at the resolution of 4 cm^−1^ using a Bruker TENSOR27 IR spectrometer (Ettlingen, Germany) based on the KBr disc method [17]. The total phenol contents were determined using the Folin–Ciocalteau method, with DMY as the standard, and expressed as DMY equivalents.

### 2.3. Determination of the Antioxidant Activity of the Covalent Complex

The antioxidant activities of the DMY, α-La, and their covalent complexes were evaluated based on the ABTS-radical-scavenging assay and reducing power measurements [18]. For the ABTS test, 0.15 mL sample solutions of different concentrations were mixed with 2.85 mL ABTS solution. Each mixture was kept in the dark for 10 min. Then, its absorbance at 734 nm (A_1_) was measured. The absorbance of the control solution (A_0_) was also determined by replacing the sample solution with water. The ABTS-scavenging activity was determined using the following equation:

(1) $$\mathrm{ABTS}\;\mathrm{radical}\;\mathrm{scavenging}\;\mathrm{activity}\;(\%)=\frac{A0-A1}{A0}\times 100\%$$

For the reducing power measurements, sample solutions of different concentrations (0.5 mL), potassium ferricyanide solution (2.5 mL, 1%) and phosphate buffer (2.5 mL, 0.2 mol/L, pH 6.6) were mixed and kept for 20 min at 50 °C. The cooled solution was mixed with 2.5 mL of 10% trichloroacetic acid solution. The mixture was centrifuged for 10 min at 3000 rpm. Then, 2.5 mL of supernatant was proportionately mixed with 0.5 mL of 0.1% ferric trichloride solution for 10 min. Then, its absorbance was read at 700 nm to represent the reducing power [19].

### 2.4. Measurement of the α-Glucosidase Inhibitory Activity of the Covalent Complex

According to a published method [20], 1 mL of sample solution (DMY, C-1, C-2, and C-3) at different concentrations was mixed with the α-glucosidase solution (1 mL, 0.2 U/mL) and kept for 10 min at 37 °C. Then, the pNP-G solution (1 mL, 1 mM) was added. The resulting solution was incubated for 20 min at 37 °C. Finally, the reaction was terminated by adding 1 mL ethanol. The absorbance of the obtained solution at 405 nm was determined as A_1_. For the control, 1 mL PBS was used instead of the sample solution, and the operation was the same as above to determine the absorbance (A_0_). The α-glucosidase inhibitory activity was calculated using the following equation:

(2) $$\alpha \mathrm{-Glucosidase}\;\mathrm{inhibitory}\;\mathrm{activity}\;(\%)=\frac{A0-A1}{A0}\times 100\%$$

### 2.5. Preparation of the Nanoemulsion Based on the Covalent Complex

DMY (0, 0.01, 0.025 and 0.05 g) and α-La (0.5 g) were dissolved in 90 mL water, stirred at 25 °C for 2 h and kept overnight at 4 °C. In order to prevent microbial growth, sodium azide was also added to the final concentration of 0.005%. With the obtained mixture as a water phase and the MCT containing 2 mg/mL β-carotene as an oil phase, the primary emulsion was prepared at the oil-to-water ratio of 1:9 via high-speed shear (15,000 rpm, 3 min). The emulsion was further homogenized at 300 bar and 4 °C 3 times using an AH-BASIC-10L/H high-pressure homogenizer (ATS, Toronto, ON, Canada), and four kinds of nano-emulsions, namely, α-La-Emulsion, C-1-Emulsion, C-2-Emulsion and C-3-Emulsion, were obtained.

### 2.6. Stability and β-Carotene-Protective Capacity of the Nanoemulsion

The β-carotene nano-emulsion was placed 15 cm under a 6 W UV lamp and incubated at 35 °C. It was sampled regularly. The corresponding β-carotene retention rate could also be determined with a UV spectrophotometer, according to the report of Li et al. [21]. Its droplet size and ζ-potential were also analyzed to reflect the stability of the emulsion using a Malvern Nano-ZS particle analyzer (Worcestershire, UK).

### 2.7. Statistical Analysis

The experimental results were expressed as the mean ± standard deviation (*n* = 3). Origin 8.0 software was applied for the statistical analysis.

## 3. Results and Discussion

### 3.1. Characterization of the Covalent Complex

**Figure 1:**
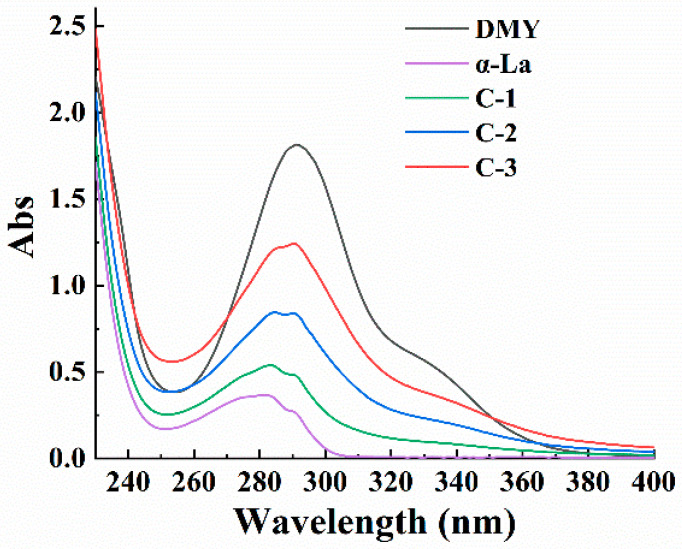
UV spectra of DMY, α-La and their covalent complexes.

The UV spectra of the DMY, α-La and their covalent complexes are shown in Figure 1. The DMY belongs to flavonol and has a characteristic UV absorption peak at 290 nm. The α-La contains a certain amount of aromatic amino acid residues (tyrosine and tryptophan); thus, there is a maximum absorption peak at 280 nm. When α-La covalently combined with DMY, the intensity of the maximum UV absorption peak of the complex rose with the increasing usage of DMY, and the increase in C-3 was the most obvious. In addition, the characteristic absorption peak of the complex was the same as that of DMY, suggesting that the basic skeleton of DMY was still retained. The shift in the maximum absorption peak of the complex indicated that the phenol hydroxyl groups of DMY were substituted.

**Figure 2:**
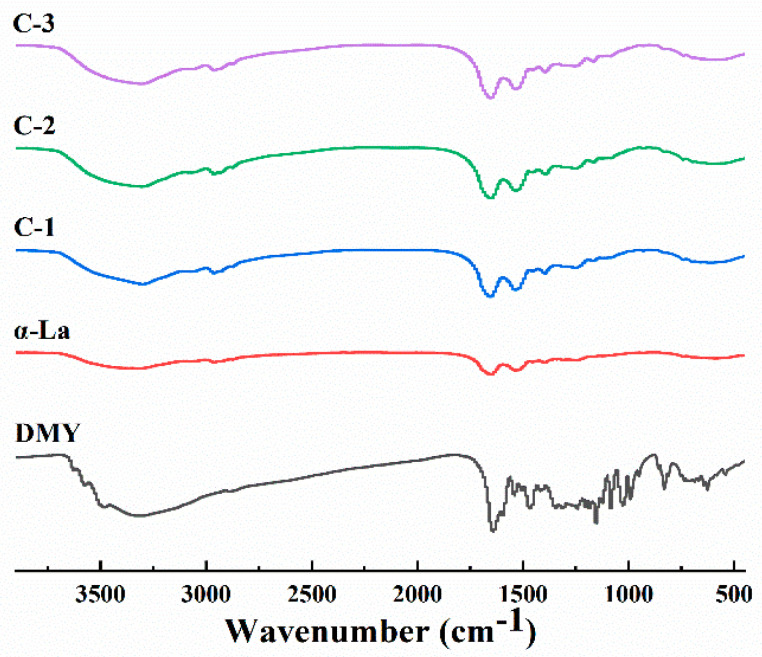
FT-IR spectra of DMY, α-La and their covalent complexes.

Figure 2 demonstrates the FT-IR spectra of the DMY, α-La and their covalent complexes. The characteristic peak of α-La at 3313.86 cm^−1^ was caused by the stretching vibration of a hydrogen bond and N-H bond, the peak at 1649.90 cm^−1^ is from the stretching vibration of a C=O bond, and the peak at 1536.60 cm^−1^ is from a C-N stretching vibration and N-H bending vibration. The DMY had the strong absorption peaks at 3329.61 cm^−1^ (phenolic hydroxyl group), 1640.41 cm^−1^ (C=O), 1154.25 cm^−1^ (C-H) and 1084.52 cm^−1^ (C-O), respectively. Compared with the IR spectrum of α-La, the IR spectrum of the covalent complex clearly changed, and the characteristic peak of C=O shifted from 1649.90 cm^−1^ to 1656.21 cm^−1^, which confirmed the covalent binding of DMY to α-La. The IR spectrum of the complex largely exhibited the characteristics of α-La, confirming that the complex was mainly composed of α-La.

The total phenol contents of C-1, C-2 and C-3 were also measured (Table 1) and could reflect the amount of grafting of DMY on α-La. The total phenol contents of α-La, C-1, C-2 and C-3 were 35.78, 42.75, 62.34 and 82.66 mg/g, respectively. The grafting amount of DMY rose with the increasing amount of DMY added during preparation. Liu et al. found a similar phenomenon in their study of a DMY-sugar beet pectin covalent polymer [22].

### 3.2. Antioxidant Activity of the Covalent Complex

**Figure 3:**
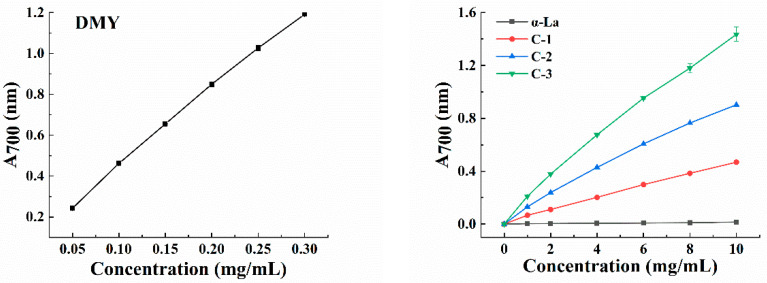
Reducing power of DMY, α-La and their covalent complexes.

Polyphenols can play an antioxidant role through donating hydrogen, donating electrons, chelating metals and in other ways [23,24]. When polyphenols are bound to proteins, the antioxidant activity of their covalent complexes is usually higher than that of their non-covalent complexes [25]. The ABTS-radical-scavenging capacity and reducing power of α-La, DMY and their covalent complexes were investigated to compare their antioxidant activities. In the ABTS test, the corresponding ABTS-radical-scavenging activity of all the samples increased with the increase in the sample concentration, and there was a positive correlation between them. The ability of the covalent complex to scavenge ABTS free radicals was significantly higher than that of α-La (Table 2). With the increase in the total phenol content in the complex, its ability was gradually enhanced, and C-3 showed the highest scavenging performance. Figure 3 exhibits the reducing power of DMY, α-La and their covalent complexes. The reducing power of DMY was the highest, followed by the covalent complexes and α-La. Moreover, the reducing power of the complex increased with the increase in the total phenol content, which aligned with the results of the ABTS experiment, fully indicating that the covalent binding with DMY enhanced the antioxidant activity of α-La. Abd El-Maksoud also found that the covalent combination of β-lactoglobulin and caffeic acid improved the antioxidant capacity and functional properties of β-lactoglobulin [26].

### 3.3. α-Glucosidase Inhibitory Activity of the Covalent Complex

α-Glucosidase is involved in the regulation of blood glucose levels in the body, and it is also a target enzyme in the prevention and treatment of diabetes [27]. It has been reported that DMY can inhibit α-glucosidase [28]. On this basis, this study analyzed the changes in the α-glucosidase inhibitory ability of α-La after combination with DMY. The α-glucosidase inhibitory performance of all the samples was positively related to the sample concentration, and the inhibitory performance of C-1 (IC_50_, 37.08 ± 1.29 mg/mL) and C-2 (IC_50_, 4.81 ± 0.16 mg/mL) was much lower than that of DMY (IC_50,_ 0.06 ± 0.00 mg/mL), while the inhibitory effect of C-3 (IC_50_, 1.78 ± 0.06 mg/mL) was similar to that of DMY (Table 3). This could be attributed to its high total phenol content, which endowed it with a strong ability to bind to enzyme proteins.

### 3.4. β-Carotene-Loaded Nanoemulsion Developed with the Covalent Complex

**Figure 4:**
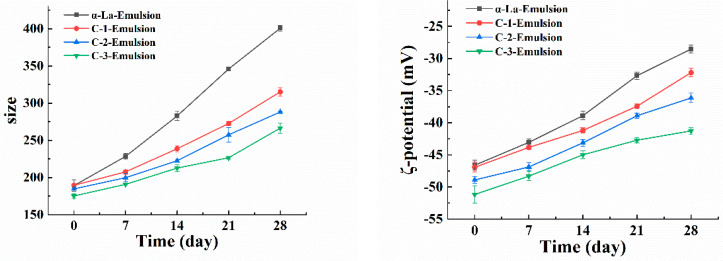
The droplet size and ζ-potential values of β-carotene-loaded nanoemulsions during storage.

Because of its excellent emulsifying properties, α-La is often applied as an emulsifier to stabilize nanoemulsions [29]. DMY has good amphiphilic and antioxidant activities [30]. Therefore, the covalent complexes of these two ingredients have both emulsifying capacity and antioxidant activity and can thus be used in the preparation of nano-emulsion. As shown in Figure 4, the droplet size and potential values of the β-carotene-loaded nano-emulsions prepared with α-La C-1, C-2, and C-3 were evaluated. Compared with the α-La-Emulsion, the emulsion prepared with the complex possessed a lower droplet size. Moreover, the droplet size declined with the increase in the total phenol content of the complex (Figure 4). When stored for 28 days, the droplet size of the emulsion stabilized with the covalent complex was less than 300 nm, while that of the α-La-Emulsion reached 400 nm. It could be concluded that the total phenol content of the complex had an obvious effect on the formation of the nanoemulsion. During the 28-day storage period, the ζ-potential value of the α-La-Emulsion also changed drastically, from −46.61 mV to −28.53 mV, (Figure 4), which suggested that the emulsifying ability of α-La itself was not enough to maintain the nanoemulsion for a long time, and when it was covalently combined with the appropriate DMY, the obtained nano-emulsion could maintain longer-term stability.

**Figure 5:**
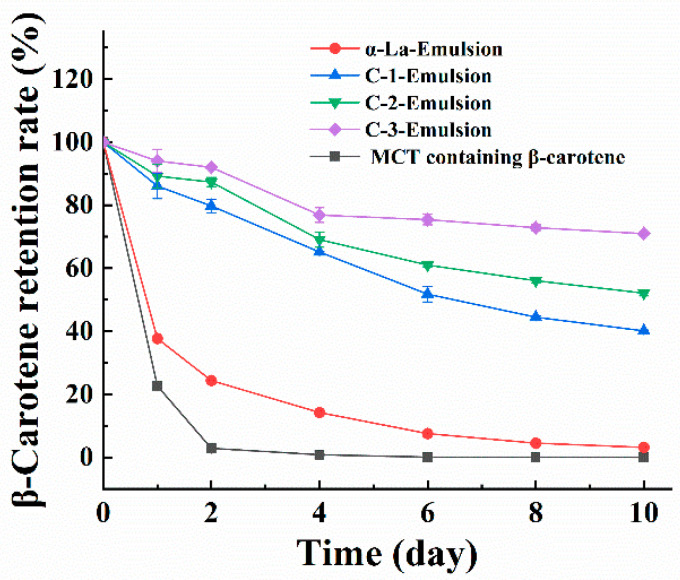
The β-carotene retention rate of nano-emulsions under UV irradiation.

Previous studies have shown that when UV irradiation is introduced, the emulsion system produces more free radicals, which accelerates the degradation of β-carotene [31]. Figure 5 demonstrates the protective effect of the nano-emulsion system on β-carotene. Compared with the control, all the nano-emulsions possessed a certain protective ability. On the second day of UV irradiation, the β-carotene in the control degraded rapidly, and its content was almost zero. At this time, the β-carotene content of the α-La-Emulsion was more than 20%, which was due to the weakening of UV irradiation as a result of the shielding effect of α-La at the O/W interface. However, the β-carotene contents of the nano-emulsions based on the covalent complexes were more than 80%. On the tenth day, the C-2-Emulsion and C-3-Emulsion could still retain more than 50% of their β-carotene, indicating that the β-carotene-protective performance of the emulsions was related to the total phenol content of the complex, mainly resulting from the antioxidant and UV absorption properties of the grafted DMY [32].

**Figure 6:**
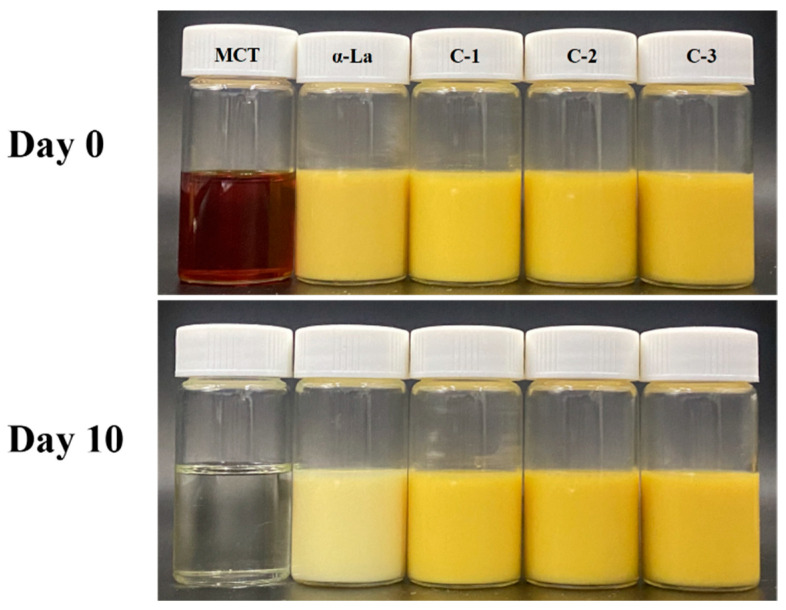
Appearance of β-carotene-loaded nano-emulsions under UV irradiation at 35 °C.

As shown in Figure 6, the nano-emulsion constructed with α-La had obviously faded after UV irradiation at 35 °C for 10 days, while the appearance of the nano-emulsions constructed with covalent complexes was better, a finding which was consistent with the determination of the β-carotene retention rate.

## 4. Conclusions

An α-La/DMY covalent complex was prepared using the alkaline method, and its structure was characterized via UV and IR analysis. Its total phenol content, α-glucosidase inhibitory capacity and antioxidant performance were positively related to the usage of DMY, added in the preparation process. The covalent complex could stabilize a stable β-carotene-loaded nano-emulsion. Its stability and β-carotene-protective capacity were higher than those of the emulsion developed with α-La and were proportional to the total phenol content of the complex. Our results could promote the application of α-La and DMY in functional foods.

## Figures and Tables

**Table 1:** Total phenol contents of α-La, DMY and their covalent complexes.

Sample	Total Phenol Content (mg/g)
α-La	35.78 ± 0.27 ^a^
C-1	42.75 ± 0.31 ^b^
C-2	62.34 ± 0.47 ^c^
C-3	82.66 ± 1.01 ^d^

Different lowercase superscript letters in the same column indicate significant differences (*p* < 0.05).

**Table 2:** ABTS-scavenging activities of DMY, α-La and their covalent complexes.

Sample	IC_50_ (mg/mL)
DMY	0.03 ± 0.00 ^a^
α-La	58.37 ± 0.52 ^e^
C-1	4.35 ± 0.10 ^d^
C-2	1.72 ± 0.02 ^c^
C-3	1.05 ± 0.01 ^b^

Different lowercase superscript letters in the same column indicate significant differences (*p* < 0.05).

**Table 3:** α-Glucosidase inhibitory activities of DMY, α-La and their covalent complexes.

Sample	IC_50_ (mg/mL)
DMY	0.06 ± 0.00 ^a^
C-DMY/α-La-1	37.08 ± 1.29 ^c^
C-DMY/α-La-2	4.81 ± 0.16 ^b^
C-DMY/α-La-3	1.78 ± 0.06 ^a^

Different lowercase superscript letters in the same column indicate significant differences (*p* < 0.05).

## Data Availability

The data are contained within the article.
